# Engaging stakeholders and target groups in prioritising a public health intervention: the Creating Active School Environments (CASE) online Delphi study

**DOI:** 10.1136/bmjopen-2016-013340

**Published:** 2017-01-13

**Authors:** Katie L Morton, Andrew J Atkin, Kirsten Corder, Marc Suhrcke, David Turner, Esther M F van Sluijs

**Affiliations:** 1 MRC Epidemiology Unit and UKCRC Centre for Diet and Activity Research (CEDAR), University of Cambridge, Cambridge, UK; 2 Centre for Health Economics, University of York, York, UK; 3 Norwich Medical School, University of East Anglia, Norwich, UK

**Keywords:** public involvement, stakeholder engagement, Delphi, physical activity, adolescent, school

## Abstract

**Objectives:**

Stakeholder engagement and public involvement are considered as integral to developing effective public health interventions and is encouraged across all phases of the research cycle. However, limited guidelines and appropriate tools exist to facilitate stakeholder engagement—especially during the intervention prioritisation phase. We present the findings of an online ‘Delphi’ study that engaged stakeholders (including young people) in the process of prioritising secondary school environment-focused interventions that aim to increase physical activity.

**Setting:**

Web-based data collection using an online Delphi tool enabling participation of geographically diverse stakeholders.

**Participants:**

37 stakeholders participated, including young people (age 13–16 years), parents, teachers, public health practitioners, academics and commissioners; 33 participants completed both rounds.

**Primary and secondary outcome measures:**

Participants were asked to prioritise a (short-listed) selection of school environment-focused interventions (eg, standing desks, outdoor design changes) based on the criteria of ‘reach’, ‘equality’, ‘acceptability’, ‘feasibility’, ‘effectiveness’ and ‘cost’. Participants were also asked to rank the criteria and the effectiveness outcomes (eg, physical activity, academic achievement, school enjoyment) from most to least important. Following feedback along with any new information provided, participants completed round 2 4 weeks later.

**Results:**

The intervention prioritisation process was feasible to conduct and comments from participants indicated satisfaction with the process. Consensus regarding intervention strategies was achieved among the varied groups of stakeholders, with ‘active lessons’ being the favoured approach. Participants ranked ‘mental health and well-being’ as the most important outcome followed by ‘enjoyment of school’. The most important criteria was ‘effectiveness’, followed by ‘feasibility’.

**Conclusions:**

This novel approach to engaging a wide variety of stakeholders in the research process was feasible to conduct and acceptable to participants. It also provided insightful information relating to how stakeholders prioritise interventions. The approach could be extended beyond the specific project to be a useful tool for researchers and practitioners.

**Strengths and limitations of this study**

- We outline a modified online Delphi process that allowed us to engage with multiple stakeholders (including young people) in the process of prioritising interventions for the Creating Active School Environments (CASE) project.
- This is the first example of Delphi techniques being used to prioritise interventions for delivery and engaging young people alongside other key stakeholders in this decision-making process.
- There was a lack of evidence for the interventions that we included in this process due to a lack of intervention research in this topic area—therefore, stakeholders relied on their own experience and judgement when making prioritisation decisions.
- The Delphi approach did not include a discussion phase in between rounds; this may have led to different outcomes had we included this.

## Introduction

There is a growing recognition of the importance of public involvement in health research, and it is now UK Department of Health policy^1^ ^2^ as well as a pre-requisite for many funding bodies.^3^ INVOLVE, a national advisory body funded by the National Institute for Health Research (NIHR), defines public involvement in research as research being carried out ‘with’ or ‘by’ members of the public rather than ‘to’, ‘about’ or ‘for’ them.^4^ This includes (but is not limited to) members of the public working with funders to prioritise what research is commissioned, offering advice as members of a project steering group; commenting on and developing research materials, and undertaking interviews with research participants.

Within public health research and practice, the term ‘stakeholder engagement’ is also frequently used. This term encompasses the ‘public’ and those served or affected by a programme or initiative, along with those involved in programme delivery and those who intend to use the results (ie, commissioners).^5^

Collaboration between researchers and stakeholders (including the public) is the best way to generate evidence that is perceived to be trustworthy and relevant by said stakeholders. This may facilitate the process of translating research evidence into practice and policy.^4^ ^6^ The growing interest in, and requirement for, public involvement and/or stakeholder engagement across all phases of the research cycle, from grant application to dissemination, means that there are multiple ways to take on board stakeholder perspectives.^4^ Target group and stakeholder engagement in intervention prioritisation values the knowledge, insights and experiences of those who are either involved in, or potentially affected by, the implementation of interventions. However, there is no consensus on how this should be achieved. This poses a challenge for researchers and practitioners who are advised (and often required) to engage stakeholders in this process. This lack of guidance can result in poorly designed and tokenistic efforts performed on an ad hoc basis.^7^

Within the context of our Creating Active School Environments (CASE) project (described in more detail below), we sought to actively engage a variety of stakeholders (defined here as ‘individuals and organisations that have a direct interest in the process and outcomes of a project, research or policy endeavour’^8^) in the intervention prioritisation phase of the research cycle. ‘Intervention prioritisation’ was the third phase of the CASE project, following evidence reviews^9^ ^10^ and secondary data analyses.^11–13^ Intervention prioritisation requires judgement based on available evidence to decide which interventions are taken forward to the subsequent phase of research (ie, carried through to ‘feasibility testing’). Usually, this step in the research process is not reported or discussed in research outputs, with researchers appraising the evidence and having the final say on which intervention makes the cut. However, as the available evidence is scarce for school environmental interventions for adolescent populations in secondary school,^9^ enlisting the knowledge and expertise of stakeholders and target groups in this process was highly relevant. Given the lack of clear guidelines or consensus on how to do this, we outline in this paper our modified ‘Delphi’ approach that allowed us to engage with multiple, geographically spread, stakeholders (including young people) to prioritise the most promising environmentally focused physical activity promotion strategies in secondary school.

### Context for the current study: CASE project

The CASE project, funded by the UK Department of Health Policy Research Programme (PRP), started in May 2014 and seeks to identify ways in which the school environment might influence adolescents’ physical activity and sedentary behaviour. The aim of this project is to identify what strategies would be most effective, acceptable and provide the best value for money for promoting physical activity and/or reducing sedentary behaviour. Within CASE, a broad definition of the school environment is applied—encompassing aspects of the school's physical (eg, classroom design and outdoor space), social (eg, teacher behaviours) and policy environment (eg, rules that influence physical activity).^14^

Throughout CASE, public involvement has been central to project progress. Two public advisory panels, who have provided input into all project phases, were established at project initiation in 2014. One consists of teachers, head teachers and parents of teenagers, and the other comprises secondary school students (aged 12–17 years). Meetings are held twice yearly. We have also compiled a list of additional CASE stakeholders, including other secondary school teachers and head teachers who are not involved in a public advisory role, academics, commissioners and practitioners with an interest in healthy schools, and various educational agencies (Department of Education, Education Funding Agency (EFA), regional schools commissioners, etc). At project initiation we additionally established a project oversight committee, the Strategic Advisory Group, consisting of an independent chair (an academic in public health) and six members, including two public involvement representatives (head teacher and parent). The principal investigator (and co-investigators where relevant) also attended the Strategic Advisory Group meetings.

All of the aforementioned stakeholders and target groups potentially have different priorities and criteria when it comes to making decisions about what interventions should be put in place. For example, ‘cost’ of the proposed intervention might be more important than ‘feasibility’ for public health commissioners. For students, ‘acceptability’ might hold more weight than ‘cost’. With multiple stakeholders and different priorities and agendas, our focus for CASE was to find a method that allowed us to engage with multiple stakeholders in order to make decisions about what intervention ultimately to implement.

### Delphi methods as a technique for engaging stakeholders in intervention prioritisation

The Delphi method is used for reaching consensus or priority setting when there is limited evidence about the topic at hand.^15–17^ Within a Delphi process, participants work independently and their contributions are anonymous, but in each round participants are provided with summary feedback from previous rounds. The process aims to reveal convergence of opinions and identify conflicting views. Its main characteristics (anonymity of participants, iteration, controlled feedback and statistical group response) allow participants to give their opinion freely, change it after having received feedback and assure that the opinion of every contributor is equally represented in the results.

Conducting a Delphi process online allows people who are spread geographically to contribute to the decision-making process, meaning that it is less costly than face-to-face expert panels.^18^ It also ensures that all participants have an equal voice in the outcome because they do not experience the interpersonal dynamics that occur in an in-person meeting.^19^ This is particularly important where different stakeholders may hold different ‘status’, as is the case in our project.

Examples of online Delphi studies do exist,^18^ ^20^ but we are unware of examples of this approach being used in the context of intervention *prioritisation* (ie, deciding what interventions are actually trialled or implemented in practice). Furthermore, there are no examples of young people being actively engaged in a Delphi study alongside other stakeholders. Therefore, the acceptability and feasibility of this approach is not known.

### Objectives of current paper

The objective of this paper is to describe our methods and critical reflections on the conduct of an online Delphi process in the CASE project. The Delphi study was employed as a means of engaging multiple stakeholders in the prioritisation of school environment-focused interventions to promote physical activity.

## Methods and results

**Figure 1:**
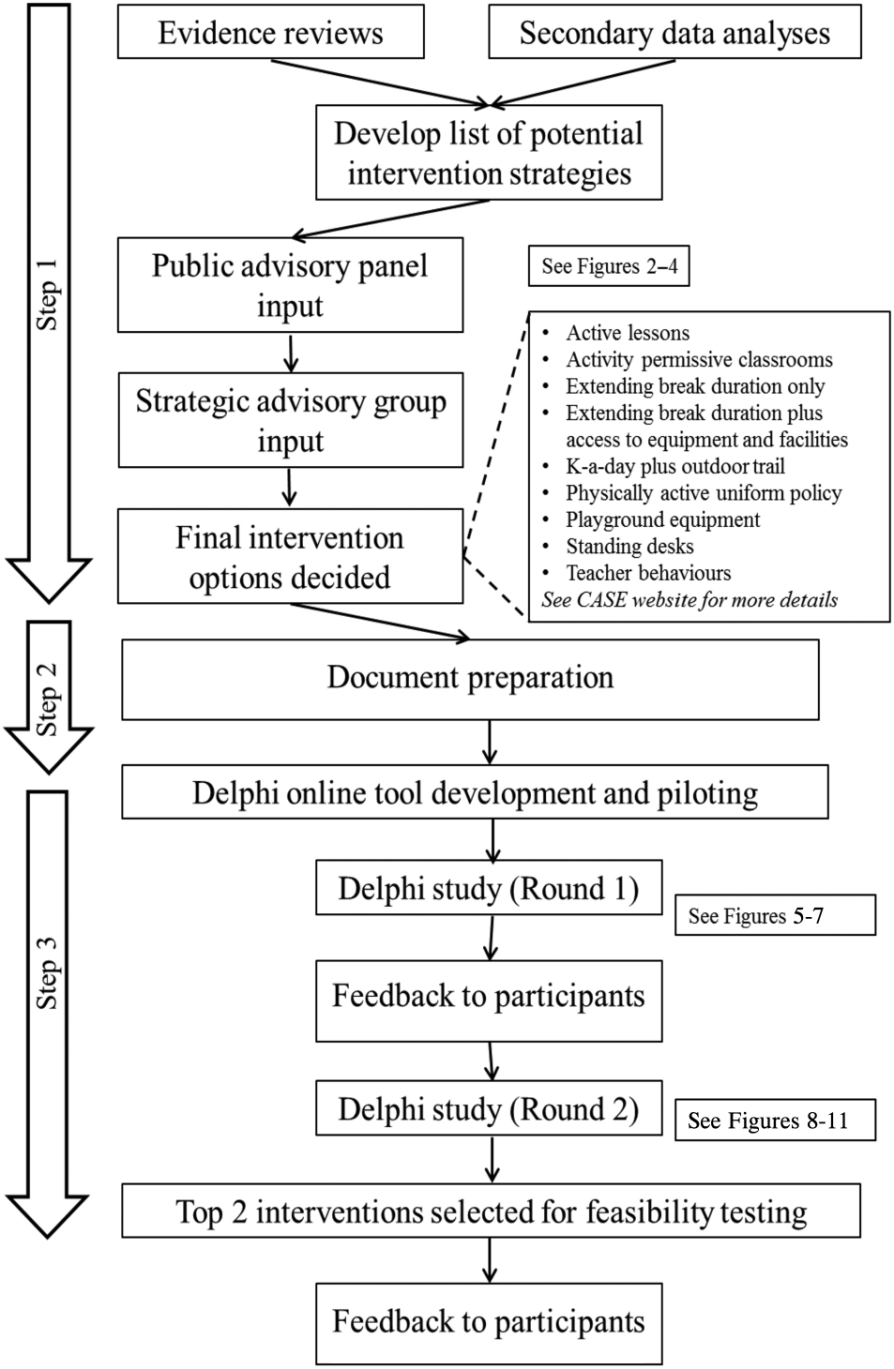
Overview of the CASE study intervention selection process. CASE, Creating Active School Environments

A diagram that outlines the CASE intervention prioritisation process is shown in figure 1.

### Step 1: developing the list of potential intervention strategies (June to July 2015)

The research team developed a list of 30 *potential* intervention strategies within a CASE project team meeting (KLM, EMFvS, AJA and KC). This list was informed by the findings of three prior phases of research in the CASE project: (1) a systematic review of published studies relating to school environments and adolescent physical activity and sedentary behaviour,^9^ (2) a review of the UK-based grey literature^10^ and (3) the findings from secondary data analyses that looked at cross-sectional and longitudinal associations between the school environment and adolescent physical activity and sedentary behaviours.^11^ ^12^

**Figure 2:**
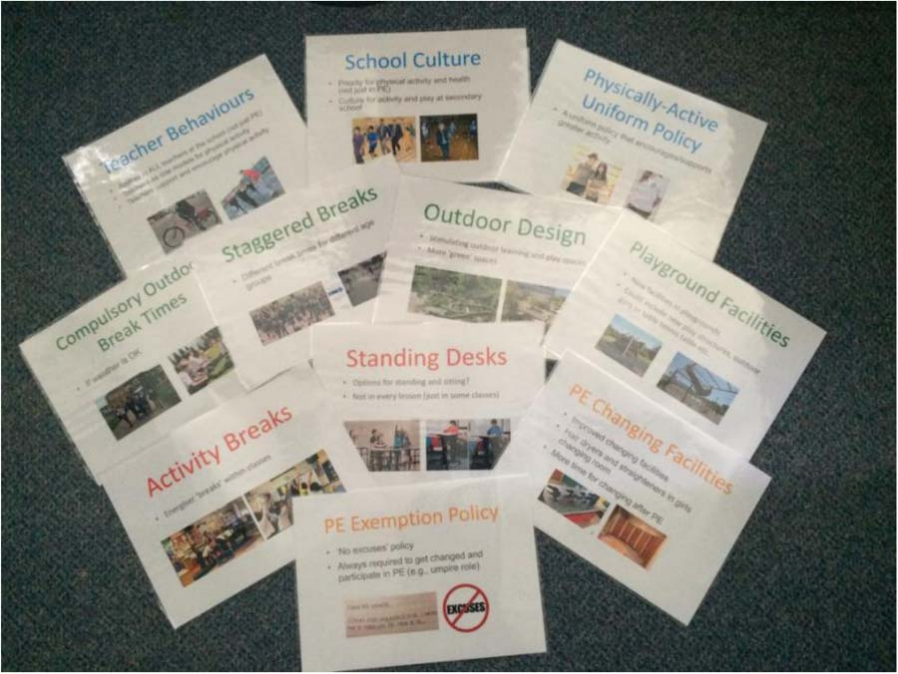
Cue cards used in the Public Advisory Group meetings. PE, physical education.

**Figure 3:**
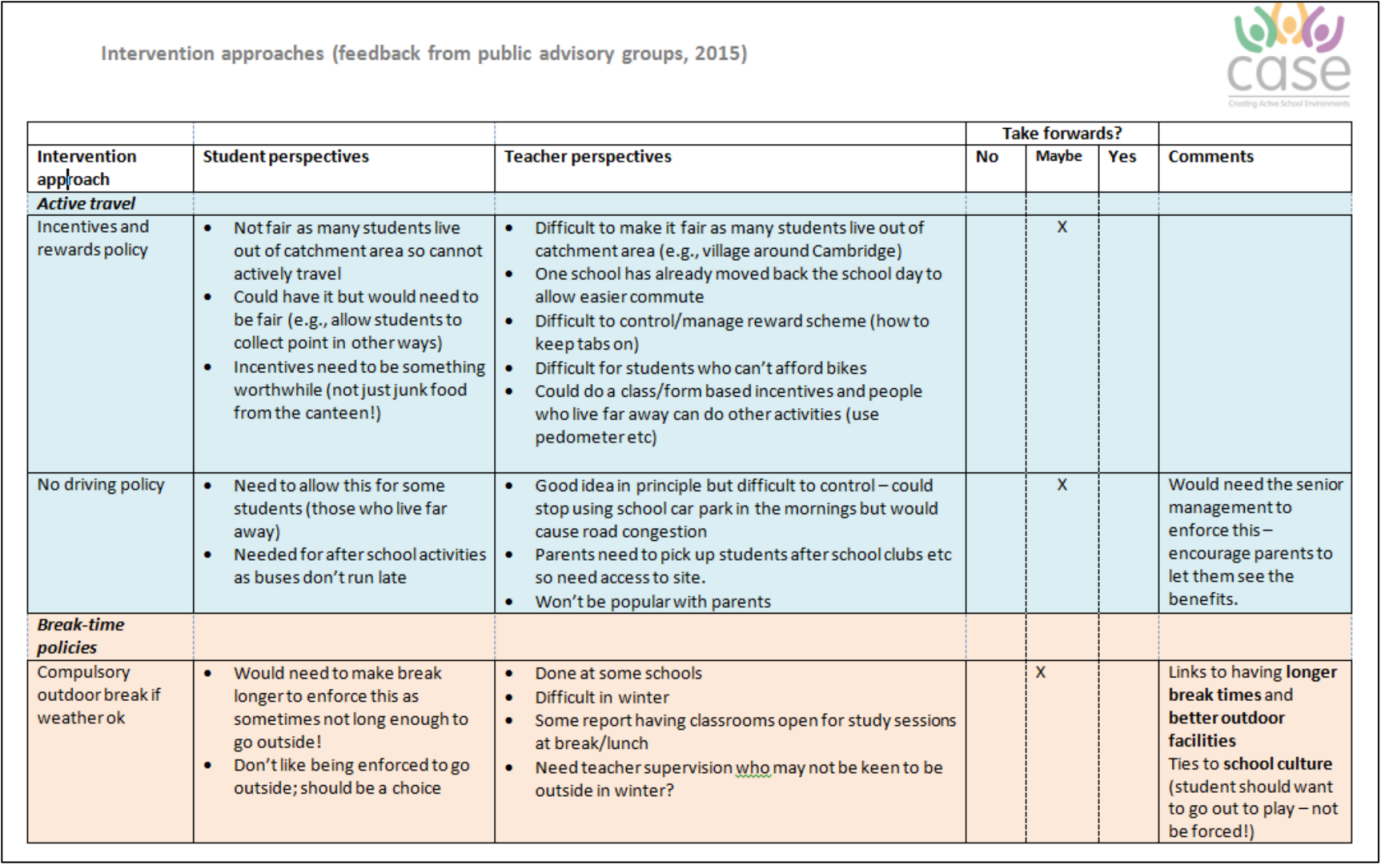
Example page from the advisory group meeting feedback summary.

**Figure 4:**
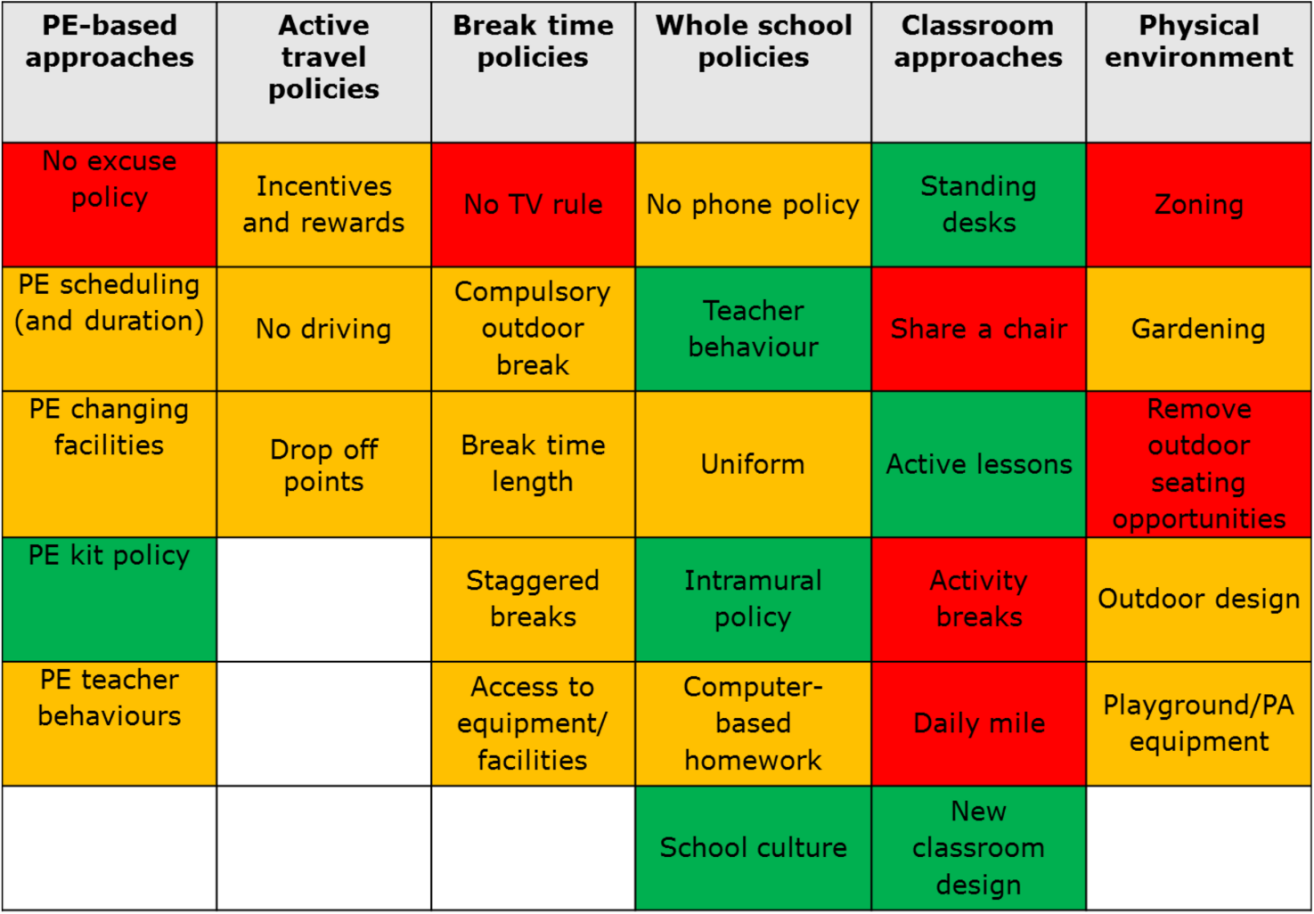
All initially proposed interventions with ‘traffic-light’ coding after Public Advisory Group meeting. PA, physical activity; PE, physical education.

The 30 candidate intervention strategies were subsequently discussed separately with our two Public Advisory Groups to obtain their recommendations for which interventions should be shortlisted for further consideration. We used cue cards to discuss each intervention in turn (see figure 2) and asked group members to decide if the intervention is placed in the ‘yes’ (green), ‘no’ (red) or ‘maybe’ (amber) pile. No predefined decision criteria were applied to enable advisory group members to express their opinions freely, and members were free to suggest additional strategies not yet included. This feedback enabled us to create a combined document that summarised the perspectives from the Young People and Adult Public Advisory Groups for each intervention (see figure 3 for an example page from this 20-page document). Figure 4 shows the combined final ‘ratings’; no/yes were only used when full consensus was reached between participants, both within and between groups.

Per prespecified protocol, a full list of the intervention strategies along with the feedback from the Public Advisory Groups was presented to our existing Strategic Advisory Group (July 2015) who made the final recommendation on which eight intervention strategies should be taken forward for further evaluation (in order to keep the prioritisation exercise manageable for participants). Again, no predefined decision criteria were applied or enforced, and the research team had no role in the decision-making process. Following this, the Strategic Advisory Group recommended nine potential intervention strategies to be considered in the intervention prioritisation project (Delphi) described here.

### Step 2: document preparation (August 2015)

Detailed documentation was prepared describing each proposed intervention strategy along with available evidence on the six prespecified criteria for decision-making.^21^ These included: reach (number of students likely to be exposed), equality (is intervention exposure and effect independent of individual characteristics), acceptability (to students, teachers and parents), feasibility (how easy the intervention is to implement), effectiveness (for physical activity promotion, enjoyment of school, academic achievement, behaviour, mental health and well-being and teacher job satisfaction) and cost.

This ‘evidence’ was a summary of findings from previous phases of research in CASE,^9^ ^10^ ^12^ augmented with feedback from the Public Advisory Group meetings and additional evidence from studies conducted in primary schools (in cases where evidence was limited for secondary school populations). There was a significant lack of experimental evidence for the majority of the proposed interventions outlined,^9^ but evidence was available from observational and qualitative studies. Where limited evidence existed, we conveyed this uncertainty and used expert judgement to outline pros and cons for each proposed intervention. Hyperlinks to published studies or other reports/news articles were added where appropriate, so that users could find more detailed information if they wished. In collaboration with our CASE Public Advisory Groups, two versions were developed for each proposed intervention—one for ‘adult’ stakeholders and one for ‘young people’. All documents are available on the CASE website: http://www.cedar.iph.cam.ac.uk/case/intervention_prioritisation_delphi/

### Step 3: Delphi study (October to December 2015)

#### Participants

We aimed to recruit ∼50 people for the Delphi study, with approximately equal representation of our three key stakeholder groups (students aged 12–17 years, education professionals, physical activity and public health professionals). Recruitment was conducted through our existing CASE stakeholders, and with public advertising in the form of a leaflet as distributed via our existing networks and advisory groups (snowball sampling) and via Twitter. Members of our Public and Strategic Advisory Groups were excluded from participation. All participants provided written (parental) informed consent prior to accessing the online system.

We received 41 positive responses, of which 37 returned a completed consent form and were provided with unique login details. Table 1 provides an overview of the recruited sample. Despite numerous invitations, we were unable to recruit representatives from Ofsted, the Department for Education (DofE) or the EFA to participate in the Delphi process.

**Table 1:** Summary of Delphi participants

Public health (N=12)	Education (N=13)	Young people (N=12)
Local authority: 5 Health-related organisations and charities: 2 Physical activity/public health-focused academics: 5	Teachers: 6 *(Maths: 1; humanities: 1; science: 1; PE: 1; SEN: 1; deputy head: 1)* School governors: 1 Regional schools commissioners: 1 Education-focused organisations/charities: 1 Academics with a focus on education: 3 Parents: 1	Secondary school students: 12 *(mean age=15.5 years; age range=13–16years)*

PE, physical education; SEN, special educational needs.

#### Delphi online tool development and pilot testing

An internally developed system facilitated the online Delphi process (more information available via the corresponding author). Participants received a unique login ID via email, enabling them to access the adult or young people version of the website as appropriate. The online Delphi process consisted of two rounds, separated by ∼4 weeks. Each round was scheduled to take no more than 30 min and responses were stored anonymously, linked to the participant's ID number. Participants could login to the online system to complete the Delphi exercises at a time and place convenient for them. No face-to-face instructions were provided. The online system was pilot-tested with five Public Advisory Group members (two teachers and three secondary school students) and their suggestions for improving instructions and making the system more user-friendly were incorporated. This included suggestions to clarify initial instructions and also changing words that were deemed too difficult to understand for most young people (eg, ‘intervention’ to ‘strategies’ and ‘implement’ to ‘put in place’).

#### First scoring round

The landing page provided instructions and details of the nine proposed interventions. Participants were subsequently guided through a series of questions, relating to the six decision-making criteria (see box 1), and asked to rank their top three intervention choices for each question. On the final page, participants were asked to rank all nine interventions in preferential order. This process enabled participants to reflect on each criterion in detail, as opposed to ranking the interventions straight away. We also asked all participants to rank the criteria (ie, cost, effectiveness, acceptability, etc) in terms of most to least important when making their overall decision. Finally, we asked them to indicate the relative importance of each ‘effectiveness’ outcome (ie, physical activity, mental health and well-being, academic achievement, enjoyment of school, concentration, behaviour and teacher job satisfaction). Box 1 lists the questions for the ‘adult participant’ version (for the young person version please visit the CASE Delphi website: http://www.cedar.iph.cam.ac.uk/case/intervention_prioritisation_delphi/).

Box 1 **Question items for each ‘criterion’**

Reach

- Which intervention will reach the most students within a school?
- Which intervention will reach the students who *need it the most*?

Equality

- Which intervention will produce effects that are unrelated to individual characteristics (eg, gender/age/disability)?
- Which intervention will produce effects (eg, increase physical activity) in those that need it most?

Acceptability

- Which intervention will be most acceptable to students?
- Which intervention will be most acceptable to teachers?
- Which intervention will be most acceptable to parents?

Feasibility

- Which intervention will be most feasible to implement for schools in the *short term*?
- Which intervention will be most feasible to implement for schools in the *long term*?

Effectiveness

- Which intervention will be most likely to increase physical activity and/or reduce sitting time for students?
- Which intervention will be most likely to improve mental health and well-being in students?
- Which intervention will be most likely to improve concentration in class?
- Which intervention will be most likely to improve students’ behaviour in school?
- Which intervention will be most likely to improve students’ enjoyment of school?
- Which intervention will be most likely to improve students’ academic achievement?
- Which intervention will be most likely to improve teachers’ job satisfaction?

Cost (effectiveness)

- The best value for money in the *short term*?
- The best value for money in the *long term*?

Finally, participants were asked to provide suggestions for modifications to the proposed interventions, additional details to be included in the evidence summaries to be considered in round 2, additional key prioritisation criteria and any additional effectiveness outcomes, and to provide overall comments. The free-text comments from the online form were reviewed to identify key issues.

**Figure 5:**
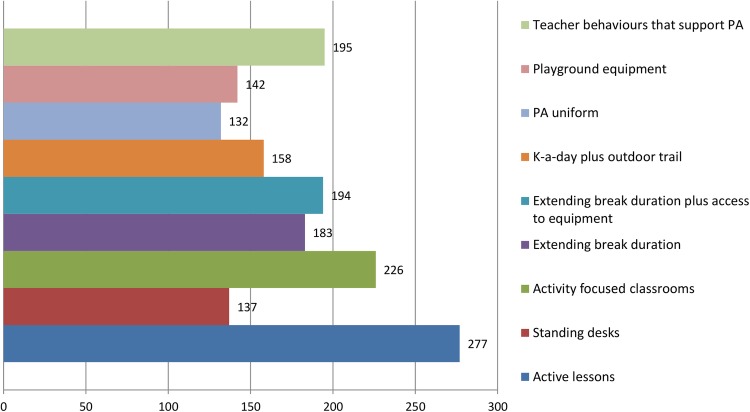
Overall scores (rankings) of the interventions after round 1. PA, physical activity.

**Figure 6:**
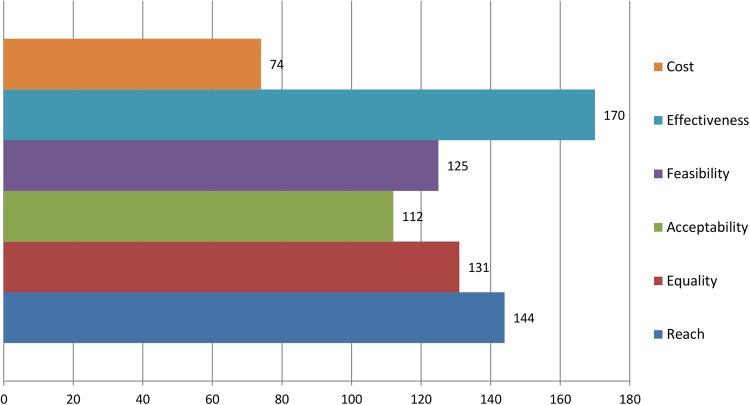
Ranking of perceived importance of each criterion (round 1).

**Figure 7:**
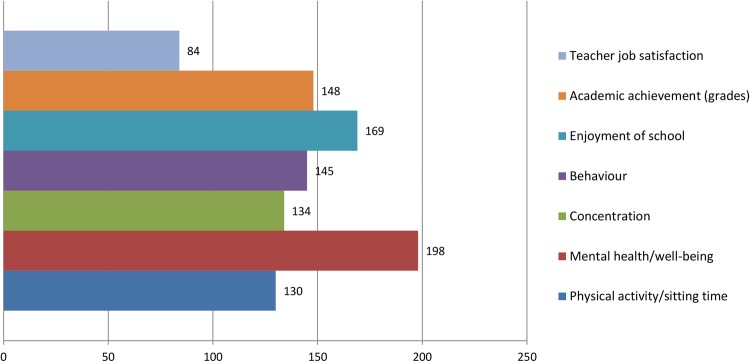
Ranking of perceived importance of each ‘effectiveness’ outcome (round 1).

Round 1 data were processed to produce a group ranking for interventions as well as the perceived importance of the prioritisation criteria. Points were allocated to reflect the respondents’ prioritisation (ie, first choice intervention=9 points, second choice intervention=8 points, etc). A total score was calculated by summing all points for each intervention. The round 1 group rankings are shown in figures 5–7. The summarised rankings for each individual item are shown in the example feedback form from round 1 (available on the CASE website: http://www.cedar.iph.cam.ac.uk/case/intervention_prioritisation_delphi/).

Sixteen participants provided free-text comments, mostly related to the cost estimates, but also raising issues regarding feasibility and acceptability, for example:

The outdoor trail costs are over estimated in my opinion. A fully sealed surface track of 1km might cost this £60k. But frankly this is over engineered; and in practice existing paths would provide at least a third of the surface. There is benefit in there being a range of surfaces to be walked on over the route; as this will benefit the feet more; and provide for a more interesting route. I think £12k—£18k all in is more likely. The construction could in parts be a project for students.

I think the active uniform is a matter of message selling to parents: a school could find active wear and traditional both acceptable. As part of wider active lifestyle behaviour messaging; this should be acceptable in all but those schools that regard the distinctiveness of the uniform as part of the school’s brand.

Comments were reviewed by the research team and added into the information available for each intervention on the online system.

#### Second scoring round

All participants received a personalised feedback document summarising the overall findings from round 1, presented next to the participant's own score (for each item). An example of this document (in full) is available on the CASE website: (http://www.cedar.iph.cam.ac.uk/case/intervention_prioritisation_delphi/). Participants were asked to access the updated online system prepopulated with their original responses. This provided them with the option to retain or change their original rankings, based on the anonymised group feedback. In total, 33 participants completed both rounds.

**Figure 8:**
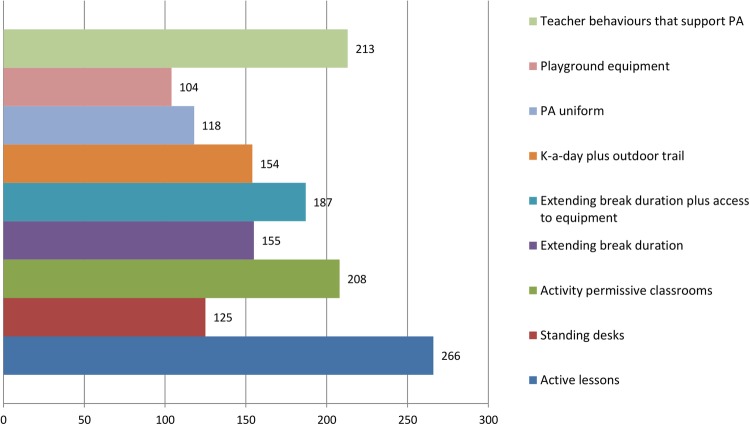
Overall scores (rankings) of the interventions after round 2. PA, physical activity.

**Figure 9:**
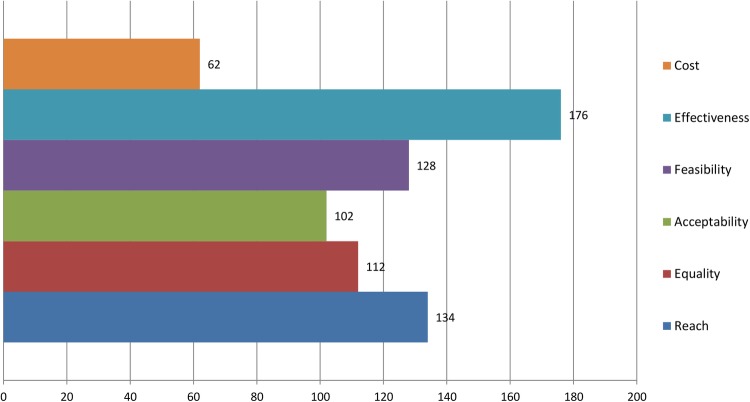
Ranking of perceived importance of each criterion (round 2).

**Figure 10:**
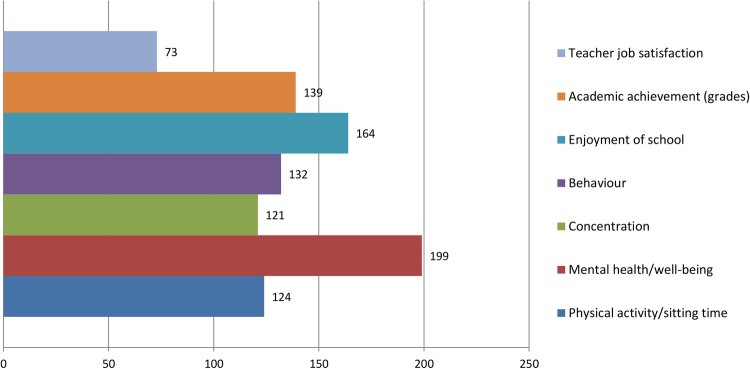
Ranking of perceived importance of each ‘effectiveness’ outcome (round 2).

**Figure 11:**
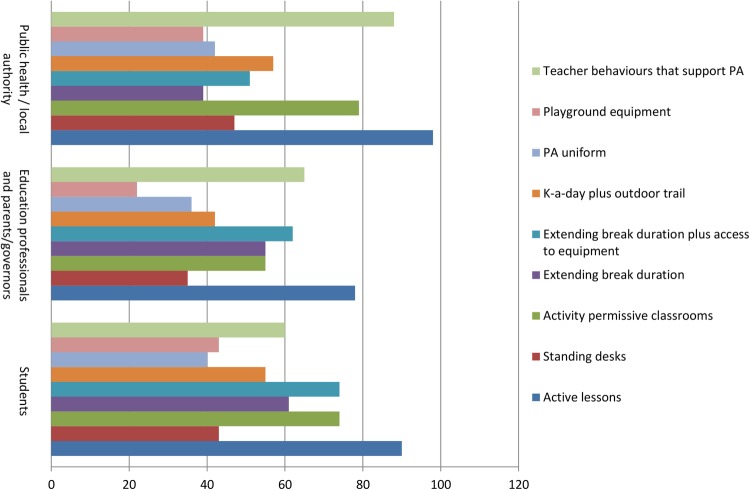
Round 2 intervention rankings, by participant group. PA, physical activity.

Results from round 2 showed increased consensus among stakeholder groups (full summary available from the CASE website: http://www.cedar.iph.cam.ac.uk/case/intervention_prioritisation_delphi/). Reviewing the overall intervention rankings showed that ‘active lessons’ was retained as the first choice overall (see figure 8), whereas shifts were observed in the second and third placed interventions. The overall group final rankings are shown in figures 8–10. Importantly, no substantial difference was observed between the various stakeholder groups in terms of the overall rankings (see figure 11). In terms of the importance of the various criteria for making the prioritisation decision, ‘effectiveness’ was consistently (in both rounds) rated the most important, followed by ‘feasibility’ and ‘reach’ (this changed slightly from round 1 to 2). Moreover, participants consistently ranked ‘mental health and well-being’ as the most important outcome to consider, followed by ‘enjoyment of school’. Interestingly, ‘physical activity’ was consistently placed at number 5 (of 6) outcomes.

### Participant feedback

A results summary was sent to all participants. We also invited participants to provide informal free-text comments on the process of being involved in the prioritisation process. Fourteen participants provided feedback; comments were generally positive, with users reporting that they found the online system ‘logical’ to use, and they found the process ‘informative’ and ‘interesting’—specifically relating to the popularity of certain interventions that they did not expect. In terms of more critical comments, a couple of users (mostly classified as public health ‘practitioners’) commented about the evidence summaries; one user commented:

The evidence cited is not referenced. This makes it difficult to make robust judgements on the interventions as the scale and scope of the related research is not known.

Finally, one user in a commissioning role explained the difficulty in justifying time for the process:

It was hard to justify the time as [name of local authority] aren't actually getting anything concrete out of the process as I assume the intervention pilots will be delivered near Cambridge.

## Discussion

This paper outlines the feasibility of the CASE approach taken to prioritise intervention strategies by engaging multiple and diverse stakeholders, including young people. Despite a substantial body of evidence on public involvement in healthcare priority setting,^7^ to the best of our knowledge, this is the first study that has used an online Delphi methodology to engage a diverse group of stakeholders in the intervention prioritisation phase of the research cycle. The results demonstrate feasibility of engaging a wide range of stakeholders to prioritise interventions using a novel online system, which ultimately guided the direction of CASE research project.

We believe that this online Delphi methodology is a feasible method of engaging stakeholders (and involving the public) in research—demonstrated specifically here for the priority setting phase of a research project. An online approach can be considered feasible if participation is relatively high—typically above 50% (of those invite actually participate), consensus is achieved, and participants are satisfied with the process.^18^ We feel that these criteria have been achieved with mostly positive comments from participants and very few drop outs between rounds 1 and 2.

Our Delphi methodology differed from other Delphi studies in that the goal was not to achieve consensus per se; rather, we sought to get insight into how different groups of stakeholders ranked the intervention options and also how they ranked the criteria on which decisions may be made. However, in all three groups of stakeholders (young people, education-focused professionals and public health professionals) the same intervention (active lessons) received the highest ranking, indicating a consensus among our stakeholders that this approach warrants further investigation. This is interesting given that active lessons are yet to be tested in adolescent populations and the evidence base provided was largely confined to studies conducted in primary schools.

The review by Mitton *et al*^7^ concluded that there is very little evidence of public involvement in the process of setting outcomes and performance measures in research. Within our Delphi study, ‘physical activity’ was not given high priority by any stakeholder group—overall, ‘mental health and well-being’, ‘enjoyment of school’ and ‘academic achievement’ were considered the most important outcomes of interest. This highlights an important point for public health researchers; specifically that other salient outcomes perceived as important by target groups must be explored and considered in addition to physical activity and physical health-related outcomes. Going forwards, we now plan to include these outcomes within the feasibility testing phase of CASE, in addition to measures of physical activity and sedentary behaviour. Outside of the research setting, this is also an important consideration for public health practitioners implementing physical activity (or health more broadly) initiatives in schools. It serves to highlight that outcomes favoured by public health advocates may differ to those considered most salient by target groups, especially schools which have many competing priorities. Changes to the way that public health initiatives in schools are marketed might be required to foster uptake and engagement with physical activity-focused initiatives.

It is tempting to speculate what direction the CASE project would have taken without this Delphi stakeholder engagement phase. In addition to the interesting (but perhaps unsurprising) finding that physical activity is not considered a top priority in terms of outcomes of interest—it may be surprising that the ‘standing desk’ intervention approach (as a standalone intervention) was ranked quite low, especially by non-physical activity/public health stakeholder groups. Given the growing popularity of this approach in primary schools and workplaces,^22–24^ the CASE research team originally anticipated the standing desk option to rank highly. We were particularly surprised by the popularity of the ‘teacher-focused’ intervention option—although the importance of teacher behaviour (reflecting wider school culture and the social environment) is widely acknowledged.^9^ ^10^ This does, however, make for a more complex intervention than we perhaps initially thought we would be implementing in the feasibility studies. The notion of researchers’ ideas being abandoned as a result of public involvement has been discussed in the literature^25^ and is an important question for researchers to ask themselves when involving the public (and stakeholders) in research.

### Challenges and limitations

The online Delphi process was not without challenges and potential limitations. One limitation was the relatively low representation of parents, and of education policymakers (eg, DofE and EFA representatives). Despite multiple attempts to engage this group, we did not recruit as many as we would have liked within the ‘education professionals’ segment. The reasons for this are unknown. This might tie to the feedback we received from a public health commissioner about a justification of time to spend on a task that one might perceive as not being directly relevant to their role (ie, public health may not be perceived by educational policymakers as their priority). This raises interesting questions about the interconnectedness of policymakers on issues relating to school and public health and potentially the (relatively) low priority given to students’ health and well-being from an education perspective.^26^

From the outset, the boundaries between ‘public involvement’ and ‘research’ were blurred. We sought ethical approval for the Delphi study, principally because we wanted to involve young people. The Ethics Board's requirement to obtain written parental consent may have acted as a barrier to recruitment for some young people. Although guidelines relating to public involvement and research ethics exist,^27^ we could not find information relating to the use of public involvement data in research publications. Clearer guidelines relating to ethical considerations for stakeholder engagement and public involvement would be welcomed to ensure that researchers make a clear distinction between ‘research’ and ‘public involvement’, especially when looking to publish the findings as we have done in the present study.

A potential limitation with our modified Delphi approach was that we did not include a ‘discussion’ phase between rounds 1 and 2. In other Delphi studies, an online forum (or similar) is established to allow participants to discuss key issues arising from round 1. We felt that this was not appropriate for our sample, given the differences in ages and professions that we included. In short, we wanted stakeholders to use their own judgement and not be ‘swayed’ by academics or practitioners who might make strong/articulate arguments in favour of their preferred intervention. Round 2 included the additional information provided by participants to enable them to see how other groups perceived the intervention strategies. It would be interesting to investigate if an online discussion phase could be implemented and whether it has any influence on the final results. Another limitation was the scoring system adopted to rank the interventions. We opted to apply a simple scoring algorithm according to how the participant ranked the interventions (ie, from 1 to 9). This assumes that the interventions are ranked linearly in a coherent order, when in fact this may not be the case (ie, individuals might find all nine relatively similar vs some might have a clear top choice and strongly dislike the others). Different online tools might allow participants to rank overall intervention preferences on a weighted scale or use a different scoring system depending on the outcome of interest. Alternative Delphi methodology (scoring systems and the use of an online forum between scoring rounds) should be considered by researchers seeking to use Delphi methods for intervention prioritisation. This would begin to establish some guidelines on the most appropriate approach.

A final challenge was the fact that there was very limited ‘evidence’ to support many of the proposed initiatives—despite the project team thoroughly reviewing the existing (un)published evidence. There was a dearth of interventions, with the majority of research exploring school environment influences being observational and/or qualitative in nature.

Furthermore, the implementation of these interventions would (in practice) be context-specific—thus making it difficult to provide evidence that applied to all cases. This is particularly true of ‘cost’ data as without evidence from actual studies about what is required to implement these interventions, the evidence provided was highly speculative and often only ‘illustrative’. We tried to make it as clear as possible that we were asking people to make a ‘judgement’ for each criterion based on their own thoughts and experiences (and using the evidence summary to provide more information that they might not have previously considered). However, some of the final comments indicated that these uncertainties complicated the ranking process.

### CASE: moving forwards

These findings will inform the decision on which intervention strategies will be considered for feasibility testing in the next phase of CASE. There is a considerable lack of useful guidance on how to combine findings from stakeholder engagement/public involvement with other forms of evidence to inform decisions about selecting interventions to trial. For CASE, solid evidence was lacking, mainly due to the fact that environment-focused interventions have largely been confined to primary schools. With this in mind, the prioritisation from stakeholder groups holds more weight and the final decision will likely reflect the findings of this Delphi study.

As reported,^7^ public involvement (and stakeholder engagement more broadly) should not be a one-off exercise. It is well documented that face-to-face contact is key^7^ and although this does not apply to our online Delphi study, within CASE we are engaging stakeholders and target group members throughout the research cycle. Our biannual Public Advisory Group meetings and other engagement activities will be key to developing interventions that are acceptable to those who will receive (or potentially commission) them.

Limited resources coupled with increasing demand for public health initiatives focused on prevention means that a process by which multiple stakeholders can be involved in commissioning decisions could be welcomed. An online process that allows for the ranking of interventions and also prioritisation of outcomes and decision-making criteria could be useful for researchers looking for public involvement in prioritisation phases of research, but also for practitioners looking to engage multiple stakeholders in decision-making. We will therefore explore the option of making this tool publically available to enable local adaptation and meet commissioners’ needs for a broad range of public health initiatives.

## Conclusions

This novel online Delphi approach to engage stakeholders in prioritising potential school environment-focused interventions was feasible to conduct, acceptable to a wide range of stakeholder groups (including young people), and provided insight into how different groups of stakeholders prioritise interventions. The use of this approach changed the proposed direction of CASE and directly influenced the intervention(s) we will trial in the next phase of the project. This online Delphi technique could be extended and applied beyond the scope of CASE to be a useful tool for public health researchers and practitioners alike.
